# AGE-RELATED CHANGES IN CUED TASK SWITCHING

**DOI:** 10.1093/geroni/igac059.2065

**Published:** 2022-12-20

**Authors:** Elizabeth Lydon, Raksha Mudar

**Affiliations:** University of Illinois Urbana-Champaign, Champaign, Illinois, United States; University of Illinois Urbana Champaign, Champaign, Illinois, United States

## Abstract

The task switching paradigm is widely used to examine cognitive switching, a critical subcomponent of cognitive control. Studies on aging suggest that switching is particularly vulnerable to age-related changes in cognition. However, the effects of manipulating the stimulus dimension on task switching performance is relatively understudied. In this study, 13 younger adults (YA; 11F; Mean Age= 22.31) and 13 older adults (OA; 8F; Mean Age= 65.85) completed a novel cued task-switching paradigm requiring speeded same-different judgments based on a perceptual (color) or conceptual (animal) dimension of the stimuli. Task switching performance was measured using switch cost, which is the difference in accuracy between switch and repeat trials. Overall both YA and OA exhibited switch costs, indicating increased cognitive demand when switching between judgments compared to repetition of the same judgement. In regard to group differences, YA and OA performed similarly when performance was collapsed across the stimulus dimensions; however, when examined separately, OA exhibited worse performance than YA when making conceptual judgements. These results highlight the importance of examining carefully manipulated stimulus-related factors in task switching paradigms to advance our understanding of cognitive control and aging.

